# A New Luminary in the East—Pointers for Osler

**Published:** 1899-06

**Authors:** 


### Editorial Department.

F. E. DANIEL, M. D., Editor.
S. E. HUDSON, M. D., Managing Editor.
A. J. SMITH. M. D., Galveston, Associate Editor.

Published Monthly at Austin, Texas, by Drs. Daniel and Hudson. Subscription price $1.00 a year in advance.

Eastern Representative: John Guy Monihan, St. Paul Building, 220 Broadway, New York City.

Official organ of the West Texas Medical Association, the Houston District Medical Association, the Austin District Medical Society, the Brazos Valley Medical Association, the Galveston County Medical Society, and several others.

### A New Luminary in the East—Pointers for Osler.

*"To the laity bright particular stars appear in the firmament about once in so often, and herald the coming of the new salvation—a cure for consumption; * * * but with the multitude in the market-place there stand scoffers, even among his own brethren, who point the critical finger at him and prove to one another how inadequate is his theory to account for the unaccountable."*—SHRADY.

*"It's better than a cirkus."*—A. WARD.

Behold, a new Light has arisen in the East; a medical and journalistic "star" of immense effulgence, which is to illumine and make clear the pathology and etiology of the great destroyer, consumption, or, as he prefers to call it, "Tuberculosis," with a capital "T." And of all the paradoxes that we ever encountered, the strangest and most provoking to a lover of harmony is that the name of this new "light" should be —"Shade!" Now, if it were "Shine," or "Shiner," or "Sit Lux," or "Hellenblazes," or even "Aurora-Borealis," it would be in keeping with the "eternal fitness of things"—and would be some comfort to a fellow. But, "Shade!" Shadow; dark; umbra; umbrageous; umbrella, in fact; that's too suggestive and altogether ghostly. Ugh! Still, it is a comfort to know that there are spots on the sun.

There are so many hum-drum editors, and so much that is either solemn or stupid or both, in the general run of medical journals now a days that it is truly refreshing to get hold of humor such as we find in the pages of a certain rather pretentious publication, with a very comprehensive and "away out yonder" name, which has lately changed owners, editors and covers, and moved east; it is edited from the nation's capital, and takes a hand in politics, as will be seen presently. It comes to us with the extraordinary claim printed on the wrapper, that it has "10,000 bona-fied subscribers." (That came very near being "ossified," didn't it?) Some of those ten thousand subscribers will be "petrified" when they read the very remarkable article with which the Journal (April number) opens. It is a new, crude and highly absurd notion of the etiology and pathology of "Tuberculosis," and a ridiculous attempt at an exposition of the same, by the editor-in-chief; and the "editorials," of which there is a "pleasing variety," all signed with the initials of the e. i. c. (that means editor-in-chief, stupid), are of the "side-splitting" kind, to a man who has any sense of the ludicrous. There is nothing so deliciously "funny" as unintentional and unconscious humor. This journal is a veritable mine of such humor. The author of the article referred to begins with the assertion that "Tuberculosis is the most considerable disease with which we have to deal"—and forthwith proceeds to "consider" it. (We learn from a "poem" elsewhere in the journal that the author of the said article and the editorials is a "well known specialist.") Like the man who put the eye in the little end of the needle and created the sewing machine, revolutionizing the sewing industry, this author reverses the order as to the cause of "Tuberculosis," upsetting and scouting all previous conceptions as to its nature, and would revolutionize its treatment. "Climate" isn't in it, a little bit; he's down on "climate."

Osler will have to revise his work on "Practice," and Koch will become a back number.

This very serious humorist says: "We really attribute the cause of infectious diseases from within the body, and not from outside causes;" and further on, announces that "it is a man's insides that undermines his constitution."

No abstract of the article—no quotations, however, will convey an adequate idea of its delicious absurdity, nor of the several editorials; and no criticism that I am capable of making will do justice to them. Hence, for the benefit of those who will be so unfortunate as to not get a copy, we reproduce a large part of the article, and some of the editorial matter, *verbatim et literatim*. We will say, however, that for disregard of orthography and syntax; for dogmatic assertion; for extraordinary liberties with the English language, which is put through a surprising course of ground and lofty tumbling, I know of nothing comparable to it. Artemus Ward convulsed the world by the use of (purposely) mis-spelled words, and (seemingly) unconscious humor; but Artemus, nor Widow Bedot, even, would not be "in it" with this blessed innocent. He says:

"We hope to have made it plain to the readers mind that we really attribute the primary cause of infectious diseases from within the body, and not from out side causes. Some conditions in acute auto-intoxication, may be brought about from exposure to the elements [sic] checking up the secretions, so that a very violent attack of acute auto-intoxication may usher in a malignant attack of any of the contagious or infectious diseases. This condition kills rapidly as in malignant Scarlet Fever, Diphtheria, Pneumonia, Meningitis, Peritonitis, Cholera Infantum, etc. But in chronic auto-intoxication we have still a greater variety of diseases developing, not from a stage of invasion as in acute diseases, but a predisposition set up in the system due to an acute attack in earlier life possibly. The principle and most important is tuberculosis, and some other wasting diseases.

"If we as a profession cling to the germ theory, which is the only rational theory, there must be a soil somewhere in the system, and it seems reasonable that this soil is peculiarly fertilized from some source, which produces a susceptibility to the germination and development of the seeds of disease sown in the prepared fields of the physical economy where the cause of suffering and death is cultivated and developed into the ripe harvests that our 'cities of the dead' demonstrate on every hand. Where is this soil, and from whence does the fertilization originate? We answer this question something in the fashion we did some time ago, when we wrote a number of articles on infections diseases, published in several Med Magazines. The soil is principally found on the inner surface of the body, and but rarely on the outer surface. We often find the budding fruitage of disease coming to the very surface of the body, and showing itself, peculiarly, depending on the nature of the infection. Many diseases cannot be diagnosed until blossoming, peculiar to its variety, appears on the surface. Too often we wait a long time or long enough for our patient to die of blood poisoning, which takes place in a number of infectious diseases, at the time or soon after the budding of its rank malignancy is discovered. We shall have more to say about acute and malignant infectious diseases later on, and try to show how to anticipate that unseen crop of germ life, and development which hides itself until it is too late to remove the fertilizing agencies which causes sudden destruction at the very first symptom that can be called diagnostic. I will undertake to show that we need not wait until the death seeds have budded and developed, before we can with mighty implements of precission remove the fertilizerwhich produces the extreme malignancy, and also sequel in the cases that happen to escape death. I refer more particularly to Scarlet, Yellow and Typhoid Fevers, including Diphtheria, Meningitis, Peritinitis, Pneumonitis, etc. But tuberculosis, which is also a deceptive and subtle disease in many respects, requires more time to develop, and the fertilizing process goes on for a longer period in many cases. So it seems to us, that when we have ample time to arrest the cultivating process, and at least retard the crop of tubercular germs that have been entertained, that heretofore were inhaled and digested with impunity, we should succeed in reducing the death rate resulting from tubercular consumption, more successfully than we have up to the present time. In regard to the soil in which the germ is fructified, which is mostly found in the muco-serous tissues of the inner surface of the body the fertilization originates from, and is wholly due to mal-assimilation and mal-nutrition, ushering in a condition of auto-intoxication, manifold forms of indigestion being forerunners of said condition of self-poisoning. We do not intend to deal with a theory that results in a phantom, but good common sense shall be our foundation, upon which we shall base our argument.

"During twenty-eight years of active practice, we have step by step arrived at conclusions which I shall allow the reader to judge, on, account of results secured by a rational plan of treatment. First of all we find the two great principals, acids and alkalies at war with each other, in throwing off excretions and secretions, and cleansing the convolutions and folds of the alimentary canal. The 'Perastalsis Muscularsis' of the intestines is handy-capped by fermentation, or chemical change, that takes place in the stomach, and thirty-five feet of intestines, so that the natural cleansing process is interfered with. When the system is alkaline, the germ of tuberculosis, are more or less entertained, and gradually the muco-serous tissues are peculiarly fertilized, so that germ life is inevitable. * * * It is the alkaline soil that entertains and develops the germs of tuberculosis with avidity, and makes the race of the many short from the cradle to the grave.

"The fermentation and decomposition in the alimentary canal, produces an unwholesome *chyle* which is carried into the right ventricle, and intermingled with the venous blood. In passing through the lungs by the law of osmosis, a considerable amount of carbonaceous and other poisoning gases are thrown off by exhalation, which fertilizes the mucus surfaces of the lung tissue, and larynx. This is why tuberculosis manifests itself more frequently in the lungs and throat, than in other parts of the body. An unwholesome chyle produces a vitiated blood. A vitiated blood produces mal-assimilation and mal-nutrition, producing a state of auto-intoxication, which is the real fertilizer in infectious diseases. This will explain why many persons are benefitted by a change of environment. The old saying that a 'change of pasture is good for sheep,' seems to hold good in the higher order of animals as well, and how very reasonable, for as soon as the digestion is restored there is no more fermentation or decomposition of the contents of the stomach and duodenum and the venous blood is replenished with a wholesome chyle, and as a result the disease is arrested, which is generally but temporary."

HARD ON TEXAS.—The author continues: "Some theorists maintain that as long as they remain away from civilization they are exempt. If this be true, it is not because there are no Tubercular germs where civilization does not exist. It is because of environment in communities or settlements, that produce a susceptibility to the entertainment and development of germ life. Such as a sedentary life a great variety of unwholesome food, and poorly cooked, hot rooms, theatres, ball rooms, billiard rooms, bar-rooms, drinking, and keeping late hours, idleness which breeds crime and consumption, constipation, indigestion, resulting in mal-assimilation, and mal-nutrition, a necessary conditions before Tuberculosis can develope.

"An active life anywhere with a few wholesome articles of food, counteract conditions in the system, that develope Tuberculosis. But coming into social and domestic life again, sleeping in close rooms, possibly too warm, attending social gatherings in warm rooms, and the going out into the night air, is more than the average constitution can bear. We are robbed of resisting power and lose our balance, or equilibrium with the elements, 'taking cold,' some call it, and the whole glandular system 'goes off on a strike.' Now, what is the result of this way of living, now in a warm room, then in a cold room, these two extremes will eventually break down and shatter the constitution of any one. But when you were in Colorado or Texas you fared quite differently, because of a different mode of living, and away from the social environment, which is to be shunned by the delicate, when it confines him to hot rooms, etc. So I contend it is not because Tubercular germs are not as plentiful in Colorado or Texas, or anywhere else you may please to mention, that you are not so easily contaminated in unsettled and sparsely populated countries, but because your mode of living and environment is different, just because you make it so by living that kind of a life, and your resisting power from the elements and environment is of such a character that you retain your physical balance, or equilibrium without any perceptible difficulty, and your assimilation of food is perfect, although you eat and drink the germs of Tuberculosis daily, you digest them with impunity, as every body does, and always has done, and always will do so long as the soil in the physical system is not peculiarly fertilized by imperfect digestion and mal-nutrition, superinduced by many causes of which I have numerated but a few. So it does not depend on the outside World, so much as it does on the inside of the body, whether we are to continue digesting all germ life we eat and drink. If we do not violate the laws of health, keeping the secretions and excretions in order, assisting nature by observing the hygienic laws in properly prepared and wholesome food, proper exercise, and also assisting nature by medicines in a wise and prudent way, then there would be much less danger of fertilizing the soil of the physical system to the entertainment and development of consumption."

Now! you fellows of the inhospitable climes; of the "effete East" —the home of consumption,—if you would get away from "civilization,"—escape the "social environment," with its "poorly-cooked, hot rooms," "theatres," "ball-rooms," "billiard-rooms," *"bar-rooms,"* and *"constipation,"*—here's your chance! Come to Texas!

A CRUCIAL (?) TEST.—This funny man says:

"To prove that Tuberculosis is not contagious, I would be willing to take a capsule of sputum for my desert every day for a month, even then the contagion theory would not cease to be agitated by certain classes who cannot help it."

CALLED HIM A HOG.—On page 77, the editor tells the following:

"In a New York street car Dr. Thos. Wilder, aged 59, asked Hugo Wolfert, aged 42, to remove his bundles from the seat and leave a lady, who was standing, sit. Wolfert refused, and the Doctor called him a 'hog,' and told him he ought to be thrown off the car Wolfert applied an epithet to Wilder, and in a moment both men, were in a lively fight, during which the doctor seriously shot Wolfert. Wilder had an ugly cut over his eye, and claimed he shot in self defense."

In the salutatory or opening editorial the editor introduces his "staff" as follows:

"It will be noticed that eight new *'Doctorea in Arte Medendi'* have been added to the Editorial Staff. Five of the eight are old class mates."

Further on (page 102), having referred to the picture and biography of one of his staff in that number, he says:

"I hereby request each member to forward their pedigree similar to Dr. Savage's in character also a photograph.—ED."

IT WAS UNCLE SAM'S TREAT.—Dropping "Tuberculosis" and taking up politics, this versatile and copious writer expresses the opinion, as well as I can make out, that if Uncle Sam had "set 'em up" for the Filipinos (or the Cubans, I can't just make out which), the war in the Philippines would have been prevented. It seems that Uncle Sam, according to the editor, "intended to treat," and notified the Cubans to that effect by proclamation, and then didn't do it; hence the war. But we will let the editor tell it:

"THE PHILIPPINES.—It seems reasonable to believe that it would have been better, had the administration issued a proclamation defining a policy as to our future plans of dealing with the Filipinos at the time of, or even before the signing of the peace treaty, by the Spanish and American peace commissioners, and not to have waited several months. During which time Filipinos have been left to themselves to imagine all sorts of diabolical things which would naturally stimulate unrest, and lead to false conclusions on their part, as to our intentions. If we intended to treat them as we notified the Cubans in a proclamation, as soon as the Island was surrendered to our forces, why did we not do likewise with the Philippines as soon as Dewey had destroyed the Spanish fleet in Manila Bay, or at least at the time a proclamation was sent to the Cubans. In fact it would have saved a great deal of blood shed, had the Filipinos been given the same particulars from the administration that has since been translated in their various dialects, and distributed throughout the Archipelago a few weeks past, after many thousands have been killed, and many homes destroyed, and war with all its horrors and devastation, from which the poor Filipinos can never recover. I have looked over many of our magazines, and find nothing on this phase of the Philippine question.

"N. B. S."

It is almost incredible that the writer of the above stuff should be a graduate of Jefferson Medical College—as I learn from the "poem" above referred to that he is,—or indeed from any medical college.

Well, this is alleged to be a free country, and—*scribimus indocti doctique.*

